# Features of Peripheral Blood Th-Cell Subset Composition and Serum Cytokine Level in Patients with Activity-Driven Ankylosing Spondylitis

**DOI:** 10.3390/ph15111370

**Published:** 2022-11-08

**Authors:** Pavel A. Shesternya, Andrei A. Savchenko, Olga D. Gritsenko, Alexandra O. Vasileva, Igor V. Kudryavtsev, Alena A. Masterova, Dmitry V. Isakov, Alexandr G. Borisov

**Affiliations:** 1 Prof. V.F. Voino-Yasenetsky Krasnoyarsk State Medical University, Ministry of Healthcare, 660022 Krasnoyarsk, Russia; 2 Federal Research Center “Krasnoyarsk Science Center”, Siberian Branch of the Russian Academy of Sciences, Scientific Research Institute of Medical Problems of the North, 660022 Krasnoyarsk, Russia; 3 Institute of Experimental Medicine, 197376 St. Petersburg, Russia; 4 Academician I.P. Pavlov First St. Petersburg State Medical University, Ministry of Healthcare, 197022 St. Peterburg, Russia

**Keywords:** ankylosing spondylitis, disease activity, T helper cells, Th17, Tfh subsets, cytokines, chemokine receptors

## Abstract

Th cells may exhibit pathological activity depending on the regulatory and functional signals sensed under a wide range of immunopathological conditions, including ankylosing spondylitis (AS). The relationship between Th cells and cytokines is important for diagnoses and for determining treatment. Accordingly, the aim of this study was to investigate the relationship between Th-cell subset composition and serum cytokine profile for patients with activity-driven AS. In our study, patients were divided into two groups according to disease activity: low-activity AS (ASDAS-CRP < 2.1) and high-activity AS (ASDAS-CRP > 2.1). The peripheral blood Th cell subset composition was studied by flow cytometry. Using multiplex analysis, serum cytokine levels were quantified and investigated. It was found that only patients with high-activity AS had reduced central memory (CM) Th1 cells (*p* = 0.035) but elevated numbers of CM (*p* = 0.014) and effector memory (EM) Th2 cells (*p* < 0.001). However, no activity-driven change in the Th17 cell subset composition was observed in AS patients. Moreover, low-AS activity patients had increased numbers of Tfh17 EM cells (*p* < 0.001), whereas high-AS activity was associated with elevated Tfh2 EM level (*p* = 0.031). The serum cytokine profiles in AS patients demonstrated that cues stimulating cellular immunity were increased, but patients with high-AS activity reveled increased IL-5 level (*p* = 0.017). Analyzing the data obtained from AS patients allowed us to conclude that Th cell subset differentiation was mainly affected during the CM stage and characterized the IL-23/IL-17 regulatory axis, whereas increased humoral immunity was observed in the high-AS activity group.

## 1. Introduction

Ankylosing spondylitis (AS) is a systemic disorder characterized by chromic injury to the paravertebral tissue, spinal joints and sacroiliac joint, with ankylosing progression occurring in the intervertebral joints and calcification developing in the spinal ligament. Currently, AS pathogenesis has not been fully elucidated. However, genetic predisposition (association with HLA-B27) and activated immune system [1,2,3] have been shown to have associations. The elevated production of some cytokines (particularly IL-17 and IL-23), which stimulate effector immune cells to markedly increase tumor necrosis factor-alpha (TNF-α) overproduction, subsequently inducing a joint inflammatory process, is recognized as one of the key mechanisms initiating this disorder [4,5,6].

AS progression is primarily linked to the development of bone tissue characterized by growing enthesophytes, syndesmophytes and/or joint ankylosis [7,8]. However, it has also been noted that high-activity AS may result from immune-related mechanisms [9,10]. Hayashi E. et al. (2016) revealed a direct association between the CD69 surface level on mucosal associated invariant T cells (MAIT cells) and AS activity [11]. Moreover, high activity AS was also coupled with elevated numbers of type 3 innate lymphoid cells (ILC3) [12,13,14]. In addition, the magnitude of the Ankylosing Spondylitis Disease Activity Score—C-Reactive Protein (ASDAS-CRP) index was found to directly correlate with peripheral blood ILC3 count. TNF-α blockers downregulate the level of mucosal addressin cell adhesion molecule-1 (MAdCAM-1) and reduce the percentage of circulating type 3 innate lymphoid cells (ILC3) [12]. It was found that high-activity AS was linked to elevated peripheral blood B cell counts and altered B cell subset composition, i.e., increased and decreased percentages of ‘naïve’ (CD19^+^IgD^+^CD27^–^) and memory B cells (CD19^+^IgD^–^CD27^+^), respectively [15,16].

Helper CD4^+^ T cells (Th) are the most abundant cellular population in the immune system. These cells may differ phenotypically, which accounts for their broad functional diversity [17,18]. Therefore, quantitative changes in Th cell functional activity have been extensively investigated in AS patients. For instance, Li M. et al. (2020) demonstrated that high-activity AS correlated with lowered levels of peripheral blood regulatory T cells (Treg), including the relevant percentage of the CD4^+^ T cell population [19]. It was found that Treg level and the Th17/Treg ratio correlated with Bath Ankylosing Spondylitis Disease Activity Index (BASDAI) magnitude [20]. Moreover, Jiang Y. et al. (2021) revealed that AS patients had elevated counts of both Th17- and Tfh-cells, and that anti-IL-17A therapy resulted in normalizing their level compared to a control group [21]. Yang M. et al. (2020) showed that patients with active-stage AS had lowered peripheral blood Th1- and Tfh1-cell counts but increased levels of Th17 (CD4^+^ T helper 17) and Tfh17 (type 17 follicular helper T cells) cells [22]. However, anti-TNF-α therapy was found to normalize the immune imbalance. At the same time, some studies have emphasized that biological disease-modifying anti-rheumatic drugs (bDMARDs) may not always result in long-term lowered disease activity, meaning that elevated AS exacerbation risk could be sustained [23]. Hence, studies are necessary to unveil alterations in diverse Th-cell compositions in patients with high-activity AS.

Effector Th cell function is executed via cytokine production and secretion, which are necessary to regulate the activity of immune and inflammatory events. It was noted that the IL-23/IL-17-cytokine axis is a major pathogenetic mechanism in AS. Therefore, AS immunopathogenesis is shaped by the activation of dendritic cells, macrophages and some other immune cells, with IL-23 production and Th17 cell activation subsequently producing IL-17 cytokines, followed by stimulated functional activity of immune cell types and the production of other pro-inflammatory cytokines [4,5,6]. For instance, Tsukazaki and Kaito (2020) noted that IL-17 stimulates macrophages, fibroblasts and other cell types to produce pro-inflammatory cytokines, which, along with IL-6, escalate in intensity in acute inflammatory processes by recruiting neutrophils to the site of inflammation [24]. In AS patients, IL-17, together with TNF-α, was found to induce a robust inflammatory cascade due to enhanced chemokine production, subsequently sustaining macrophage and neutrophil recruitment to the site of inflammation [5,25]. In addition, it should be mentioned that during such an inflammatory event, other Th cell subsets become activated, each of which bears its own cytokine set which is introduced into the developing mechanisms of inflammation in AS [20,26]. Hence, investigating cytokine levels in activity-driven AS allows us not only to characterize cytokine-related regulatory mechanisms, but also to assess functional activity in diverse Th cell subsets. The characterization of the CD4+ T cell subsets and cytokine serum levels in AS patients will allow us to understand the immune mechanisms that depend on the activity of the disease and which must be taken into account when conducting immunotherapy.

Thus, the current study investigates the features of Th-cell subset composition and serum cytokine profiles for patients with activity-driven AS.

## 2. Results

### 2.1. Assessing Peripheral Blood Th Cell Subset Composition in Activity-Driven AS Patients

While investigating the peripheral blood Th cell quantities and relevant cell subset compositions in AS patients, it was found that the percentages and absolute numbers of total helper T cells were unrelated to AS activity (Table 1). Assessing the surface CD45RA and CD62L co-expression levels revealed that this repertoire changed with Th cell maturation and the acquisition of effector cell functions, allowing us to identify a ‘naïve’ Th (CD45RA^+^CD62L^+^) subset and estimate the level of mature as well as central memory (CM), effector memory (EM) (CD45RA^–^CD62L^+^ and CD45RA^–^CD62L^–^, respectively), and terminally-differentiated effector memory CD45RA^+^ T cells (TEMRA, CD45RA^+^CD62L^–^) [27,28]. We found that only patients with low-activity AS had lowered percentages of peripheral blood CD45RA^–^CD62L^–^ EM Th cells. In contrast, high-activity AS was linked to decreased levels of peripheral blood CD45RA^+^CD62L^+^ ‘naïve’ Th cells compared to those of the control group, whereas in high- vs. low-activity AS, the levels of CD45RA^–^CD62L^+^ CM Th cells were elevated.

We also performed a detailed analysis of CM and EM Th cell composition by assessing the expression of cell-surface chemokine receptors CCR4, CCR6, CXCR3 and CXCR5. It was found that low-activity AS patients vs. the control group had elevated absolute numbers of peripheral blood CM Th, along with increased percentages of CCR6^+^ Th-cells (Table 2). In contrast, high-activity AS patients were shown to have decreased percentages of CXCR5^–^CXCR3^+^CCR6^–^CCR4^–^ Th1 cells and elevated levels of CXCR5^–^CXCR3^–^CCR6^–^CCR4^+^ Th2 cells, as well as CCR6^+^ Th17 cells, along with increased absolute numbers of CM Th, compared to the control group.

Our investigation of CM Th17 cell subset composition revealed that both low- and high-activity AS had increased percentages of CXCR5^–^CXCR3^–^CCR6^+^CCR4^+^ ‘classical’ Th17 and CXCR5^–^CXCR3^–^CCR6^+^CCR4^–^ DN Th17 cells, along with decreased levels of CXCR5^–^CXCR3^+^CCR6^+^CCR4^+^ Th17.1 cells (Table 3), compared to the control group.

The CM Tfh cell subset composition in AS patients is shown in Table 4. In particular, it was found that regardless of disease activity, patients had lowered percentages of CXCR5^+^CXCR3^+^CCR6^–^CCR4^–^ Tfh1 cells, as well as elevated quantities of Tfh2 and Tfh17 cells with CXCR5^+^CXCR3^–^CCR6^–^CCR4^+^ and CXCR5^+^CXCR3^–^CCR6^+^CCR4^–^ phenotypes, respectively, compared to the control group. At the same time, high-and low-activity AS patients were found to have decreased percentages of CXCR5^+^CXCR3^+^CCR6^+^CCR4^+^ ‘double-positive’ DP Tfh cells compared to the control group.

Chemokine receptor expression was also analyzed in EM Th-cells. It was found that low-activity AS patients had elevated percentages of peripheral blood CCR6^+^ EM Th-cells (Table 5) compared with the control group, whereas high-activity AS was characterized by an increased percentage of CXCR5^–^CXCR3^–^CCR6^–^CCR4^+^ Th2 cells compared with the low-activity AS and control groups. Our assessment of EM Th17 cell subset composition revealed that regardless of AS activity, patients had increased percentages of ‘classical’ Th17 and DN Th17 cell subsets (CXCR5^–^CXCR3^–^CCR6^+^CCR4^+^ and CXCR5^–^CXCR3^–^CCR6^+^CCR4^–^, respectively) (Table 6), compared with the control group.

However, only low-activity AS patients vs were found to have increased percentages of CXCR5^+^CXCR3^–^CCR6^+^CCR4^–^ Tfh17 cells (Table 7) compared to the control group. Furthermore, a decreased level of CXCR5^+^CXCR3^+^CCR6^–^CCR4^–^ Tfh1 cells was observed in both AS patient groups compared with the control group. Moreover, in high-activity AS patients, an increased percentage of CXCR5^+^CXCR3^–^CCR6^–^CCR4^+^ Tfh2 cells, paralleled with a decreased percentage of CXCR5^+^CXCR3^+^CCR6^+^CCR4^+^ DP Tfh cells, was observed, as compared with control and low-activity AS subjects.

### 2.2. Serum Cytokine Profile in Activity-Driven AS Patients

A multiplex assay allowed us to investigate the serum cytokine profiles of AS patients. It was found that low-activity AS patients had elevated levels of IL-2 and IL-17E/IL-25 (Table 8) compared to the other groups. At the same time, only high-activity AS patients had increased serum levels of IL-15. Regardless of the disease activity, AS patients had increased concentrations of serum IL-12(р40), IL-13, IL-17А, IL-17F, IFN-γ and MSCF, compared to the control group.

### 2.3. Features of Associations between Th Cell Subsets and Serum Cytokine Levels in Activity-Driven AS Patients

A correlation analysis was used to assess the relationship between Th cell subset composition and serum cytokine level with regard to AS activity. In particular, it was found that low-activity AS was positively associated with the percentage of CM CXCR3^–^CCR6^–^CCR4^+^ Tfh2 cells and serum concentration for IL-5 (r = 0.42, *p* = 0.041) and IL-12(p40) (r = 0.47, *p* = 0.021), whereas percentage of CM CXCR5^–^CXCR3^+^CCR6^+^CCR4^+^ DP Th17 cells correlated with IL-6 level (r = 0.44, *p* = 0.031). Moreover, in this patient group, the serum MCSF level was positively or negatively correlated with the percentage of CXCR5^+^ Th (r = 0.43, *p* = 0.034) and CM CXCR3^–^CCR6^+^CCR4^–^ Tfh17 cells (r = 0.49, *p* = 0.016) or CM CXCR5^–^CXCR3^+^CCR6^–^CCR4^–^ Th1 cells (r = –0.45, *p* = 0.026), respectively. Investigating a relationship between EM Th-cells and serum cytokine levels in low-activity AS patients revealed that the percentage of EM CXCR3^–^CCR6^–^CCR4^+^ Tfh2 cells was positively correlated with the concentration of serum IL-4 (r = 0.50, *p* = 0.014), IL-5 (r = 0.42, *p* = 0.043) and IL-6 (r = 0.50, *p* = 0.014). Furthermore, in this patient group, serum MCSF level was negatively associated with the absolute numbers of CXCR3^+^CCR6^–^CCR4^–^ Tfh1 and CXCR3^+^CCR6^+^CCR4^+^ DP Tfh cells (r = –0.43, *p* = 0.037 and r = –0.42, *p* = 0.039, respectively).

Furthermore, high-activity AS patients were found to have a positive association between serum IL-4 level and percentage of CXCR5^–^CXCR3^–^CCR6^–^CCR4^+^ Th2 cells (r = 0.77, *p* = 0.025), as well as CM CXCR5^–^CXCR3^–^CCR6^+^CCR4^+^ ‘classical’ Th17 cells (r = 0.75, *p* = 0.033). Moreover, such patients also revealed a negative correlation between the level of CXCR5^+^ CM Tfh cells and serum IL-17F (r = –0.69, *p* = 0.038), as well as IL-6 (r = –0.72, *p* = 0.030) level. Investigating the relationship between EM Th cells and serum cytokine concentration in high-activity AS patients revealed a positive association between the relative number of CXCR5^–^CXCR3^–^CCR6^+^CCR4^+^ ‘classical’ Th17 cells and serum level of IFN-γ (r = 0.81, *p* = 0.009) and IL-12(p40) (r = 0.68, *p* = 0.045). In addition, serum IFN-γ level, in turn, was positively associated with the level of CXCR5^–^CXCR3^+^CCR6^+^CCR4^+^ Th17.1 (r = 0.70, *p* = 0.037) and EM CCR6^+^ Th17 cells (r = 0.68, *p* = 0.044). Moreover, a positive correlation between the relative number of EM CXCR5^–^CXCR3^–^CCR6^–^CCR4^+^ Th2 cells and serum IL-4 level (r = 0.76, *p* = 0.029) was found.

## 3. Discussion

Developing effector Th-cell subsets undergo a two-stage maturation comprising antigen-independent thymic differentiation and antigen-dependent differentiation occurring in the secondary peripheral immune organs. The first stage results in egress of ‘naïve’ CD45RA^+^CD62L^+^ Th cells into peripheral blood, whereas stage 2 involves subsequent antigen-dependent differentiation of the central memory (CM, CD45RA^–^CD62L^+^), effector memory (EM, CD45RA^–^CD62L^–^) and terminally-differentiated effector Th cells (TEMRA, CD45RA^+^CD62L^+^) [27,29,30]. T cell differentiation may be assessed by detecting the surface CD45 isoform, a transmembrane tyrosine phosphatase-coupled protein. The extracellular CD45 portion is encoded by seven exons [31,32,33]. ‘Naïve’ Т cells are found to bear all domains in this region of CD45 molecules, being denoted as CD45RA (with a molecular weight of 220 kDa). During antigen-dependent differentiation, CD45 mRNA undergoes alternative splicing, resulting in the loss of CD45 extracellular domains to be further transformed via intermediate isoforms. TEMRA Th-cells also express CD45RA [34]. Hence, Th-cells which are negative for CD45RA are defined as memory Th-cells (CM and EM), whereas Th cell subsets expressing it are referred to as the naïve and TEMRA subsets.

Another surface molecule being expressed on Th-cells which is used for phenotyping is CD62L, an L-selectin that accounts for cell migration into peripheral lymphoid tissues by recognizing cognate endothelial ligands such as GlyCAM-1 (glycosylation-dependent cell adhesion molecule-1), MadCAM-1 (mucosal addressin cell adhesion molecule-1) and CD34 (endothelial cell protein) [35,36]. Therefore, CD62L is expressed on naïve and CM T cells (accounting for their migration into peripheral lymphoid tissues) but not on EM T cells or TEMRA (to function outside lymphoid tissues) [37].

While analyzing co-expressed the CD45RA and CD62L profiles in Th-cells in AS patients, it was found that low disease activity was associated with a decreased percentage of peripheral blood EM Th-cells, whereas high-activity AS was linked to a lower absolute number of naïve Th-cells but an increased level of CM Th-cells. Overall, it may be concluded that alterations in Th-cell phenotypes during cell differentiation in AS patients reflect the disease course. CM Th-cells are characterized as a long-living cell subset which mainly recirculates via secondary lymphoid tissues [38,39]. In contrast, EM Th-cells are mature effector cell types displaying lowered proliferative potential, even though their capacity to produce and secrete diverse cytokines, along with the expression of effector molecules, substantially exceeds those found in CM Th-cells [39,40].

Next, we conducted a detailed analysis of the CM and EM Th-cell subset composition according to the surface chemokine receptor-based expression for CCR4, CCR6, CXCR3 and CXCR5.

CCR4 is a β-chemokine (CCL2 (MCP-1), CCL4 (MIP-1), CCL5 (RANTES), CCL1 (TARC), CCL22) molecule belonging to the G-protein-coupled receptor family; it acts as a lymphocyte homing receptor [41,42]. CCR6 is another β-chemokine receptor which only interacts with CCL20 (MIP-3α); it is produced by fibroblasts and endothelial and dendritic cells [43,44]. CXCR3 belongs to the CXCR receptor family and able to sense the three CXC-chemokines, i.e., CXCL9 (MIG), CXCL10 (IP-10) and CXCL11 (I-TAC), which are produced by diverse organ and tissue cell types upon initiation, primarily triggered by IFN-γ, of inflammatory processes [45,46,47]. The CXCR5 receptor belongs to the CXC-superfamily composed of G-protein-coupled molecules binding to the chemokine CXCL13 [48,49,50].

Based on the expression for receptors CCR4, CCR6, CXCR3 and CXCR5 on Th-cells, the major polarized peripheral blood helper T cell subsets were determined: Th1- (CXCR5^–^CXCR3^+^CCR6^–^CCR4^–^), Th2 (CXCR5^–^CXCR3^–^CCR6^–^CCR4^+^), Th17 (CXCR5^–^CCR6^+^) and Tfh cells (CXCR5^+^) [51,52,53,54]. It was found that low-activity AS patients had elevated percentages of Th17-cells, along with an increased absolute number of CM Th cells. At the same time, high-activity AS patients had increased percentages of peripheral blood Th2 and Th17 cells but decreased Th1 cell levels and increased absolute numbers of CM Th cells. Th17 cells are the major source of IL-17 production; they are also able to secrete a broad cytokine and chemokine profile which primarily exerts pro-inflammatory activity. Not surprisingly, a cytokine multiplex assay in AS patient sera revealed elevated concentrations of IL-17 family (IL-17A, IL17E/IL-25 и IL-17F) cytokines; this was virtually unrelated to disease activity. Among them, IL-17A and IL-17F may act on stromal cells and lymphocyte subsets, which initiate inflammatory responses [55,56]. In addition, it was evidenced that IL-17A may mediate bone injury by inducing RANK expression on the osteoclast surface, as well as upregulating RANKL production via mesenchymal stem cells [26]. It is also worth emphasizing that elevated levels of IL-17 cytokine family members were revealed in our therapeutic interventions.

Th1/Th2 balance has been widely investigated in AS immunopathogenesis [6,19,26]. It has been shown that Th1 cells elicit an immune response against intracellular pathogens by secreting IFN-γ, which acts as a macrophage-activating cue [26,57]. Its serum concentration in the patients examined in this study was elevated, regardless of the AS activity. Apart from IFN-γ, Th1-cells are also able to produce IL-2, IL-10 and TNF-α, which are involved in inflammatory processes. However, a single negative correlation was only observed between peripheral blood CM Th1 cells and serum MCSF (macrophage colony-stimulating factor) levels. MCSF (CSF-1), bearing a core of the four α-helices, is the key cytokine, ensuring macrophage proliferation, differentiation and survival; it is produced by fibroblasts, activated macrophages, osteoblasts, activated endothelial cells as well as bone marrow stromal cells [58,59,60]. It was confirmed that osteoblast-released MCSF exerts a paracrine activity on osteoclasts that results in bone resorption [61]. It may be assumed that during AS, such a relationship characterizes a developing competitive interplay between innate and adaptive immunity, which may become evident during therapeutic intervention.

Th2 cells are important for the eradication of extracellular parasites; their cytokine profile consists of IL-4, IL-5 and IL-13 [26,57]. Hence, elevated levels of CM Th2 cells, along with lowered Th1-cell levels in high-activity AS patients, characterize a prevalence of protective type 2 immune responses. In particular, only this cohort of patients featured elevated serum IL-5 levels, which shows a positive association between Th2 cells and serum IL-4 concentration.

Four major Th17 cell subsets were identified within the total CCR6^+^ CM and EM Th cell population, which differed both in terms of the chemokine receptor CXCR3 and CCR4 expression patterns as well as the range of functional activities [62,63,64]. To date, ‘classical’ CCR4^+^CXCR3^–^ Th17-cells, double-positive CCR4^+^CXCR3^+^ Th17-cells (DP Th17 cells), non-classical CCR4^–^CXCR3^+^ or Th17.1-cells as well as double-negative CCR4^–^CXCR3^–^ (DN Th17 cells) subsets have been distinguished. ‘Classical’ Th17 cells are able to actively produce IL-17A, whereas the release of cues such as IL-22 and GM-CSF is not prominent. We observed that the related cell proliferation was less markedly suppressed by pro-inflammatory cytokines. Non-classical vs. ‘classical’ Th17 cells show higher TCR-driven proliferative potential. Th17.1 cells may produce a great quantity of GM-CSF, accounting for neutrophil recruitment and activation in the site of inflammation, whereas IFN-γ elicits tissue macrophage activation [65,66]. Moreover, Dankers et al. (2021) demonstrated that both classical and non-classical Th17 cells, together with synovial fibroblasts, may become mutually activated by generating an inflammatory loop by recruiting other pro-inflammatory immune cells and releasing matrix metalloproteases [62]. DN Th17 vs. classical Th17 cells expressed higher levels of IFN-γ, IL-17A, IL-17F, MIP-3a/CCL20 and TNF-α, whereas the potential to express the IL-13 encoding gene was reduced. It is believed that it is the CCR6^+^DN cells which largely produce IL-17F and IL-8. Moreover, DN Th17 cells express high levels of mRNA for the molecules which are responsible for lymphoid tissue migration (CCR7, CXCR5, CXCL13, SELL, SIRP1, JAM3, AIF1) [66,67]. Among all Th17 cell subsets, DP Th17 cells (CXCR5^–^CXCR3^+^CCR6^+^CCR4^+^) possess the lowest capacity to produce IFN-γ, IL-17A, TNF-α and IL-13. However, they may express adhesion molecules and chemokine receptors, ensuring cell migration into the site of inflammation [62]. We found that regardless of AS activity, the quantity of both peripheral blood CM classical and DN Th17-cells was increased, in parallel with a decline in DP Th17-cell levels. Therefore, even during therapeutic interventions, the AS patient cohort showed increased percentages of actively-cytokine producing Th17 cells, as confirmed by the multiplex assay. With this in mind, low-activity AS was associated with a positive correlation between CM CCR6^+^DP cells and serum IL-6 levels that characterize changes in the related cell counts and the relevant activity of inflammatory events. In addition, it is worth noting that cytokine IL-6 stimulates Th17 cell differentiation, resulting in an imbalance between pathogenic Th17 and regulatory T cell subsets [68]. With high disease activity, a correlation between levels of classical Th17 cells and serum IL-4 was observed that further characterizes a more profound involvement of the humoral immune arm in AS immunopathogenesis under high disease activity.

Tfh cells oversee the fully controlled development of B-cell-dependent humoral immune response in germinal center follicles within secondary lymphoid tissues (B cell proliferation, plasma cell and memory B cell differentiation, immunoglobulin isotype switching, etc.). Moreover, Tfh-cells produce quite a broad range of diverse cytokines, among which the major cues are IL-10 and IL-21 [69,70,71]. An approach based on assessing surface CXCR3 and CCR6 chemokine receptor expression was used to analyze the composition of the Tfh-cell subset [72]. Owing to this, a whole set of human circulating peripheral blood Tfh-cells was divided into four distinct subsets: Tfh1-cells (CXCR3^+^CCR6^−^), Tfh2-cells (CXCR3^−^CCR6^−^), Tfh17-cells (CXCR3^−^CCR6^+^) and dual-positive Tfh-cells (DP Tfh-cells, CXCR3^+^CCR6^+^). Tfh1 cells (CXCR5^+^CXCR3^+^CCR6^–^CCR4^–^) share some Th1 cell properties, including CXCR3 expression (in the absence of CCR6 surface expression), as well as a potential to produce IFN-γ in response to functional stimuli [73,74]. Furthermore, it was revealed that such Tfh1 cells down-regulate antibody production by ‘naïve’ B cells in vitro [72]. In contrast, Tfh2 cells have similarities with Th2 cells, in particular, the expression of transcription factor GATA3 and the coupled production of the IL-4, IL-5 and IL-13 cytokines. Therefore, in Tfh2 cells co-cultured with ‘naïve’ B cells, the latter enhanced production of all major antibody classes [73,75]. Similar to Th17 cells, Tfh17 cells similar to Th17 cells are able to produce IL-17A and IL-22 in parallel with expressing the transcription factor ROR t. Moreover, Tfh17 cells may also elevate in vitro antibody production by naïve B cells except IgE, so that the peak effect may be observed when IgA production is induced [71,76]. DP Tfh-cells are able to extensively produce IL-21 and IL-4 [77]. We found that regardless of AS activity, the level of peripheral blood CM Tfh1 declined in parallel with an increase in the quantity of CM Tfh2 and Tfh17 cells. It should be noted that the dominance of Tfh2 and Tfh17 cells over the Tfh1 cell subset is related to the immunopathogenesis underlying systemic and organ-specific autoimmune disorders [78]. Based on the imbalanced Tfh cell subset composition and subsequent cytokine production, a pivotal role for the immunopathogenic IL-23/IL-17-axis has been identified. This was confirmed by the positive correlation between peripheral blood CM Tfh2 cells and serum IL-12(p40) level found in low-activity AS; its concentration was elevated both in low- and high-activity AS patients. IL-12(p40) is a constituent in both IL-12 and IL-23 cytokines which is also able to act as a macrophage chemoattractant and to stimulate activated dendritic cells [79,80]. Paiva I.A. et al. (2021) noted that it is IL-12(p40) that initiates an IL-23/IL-17 cytokine cascade [81]. In addition, low-activity AS patients had levels of CM Tfh2 cells that were positively correlated with serum IL-5 concentrations which characterize functional activity of this cell type and the manifestation of humoral mechanisms during AS. The cellular arm in AS immunopathogenesis was confirmed by the positive correlation between serum MCSF level and the total CM Tfh (CXCR5^+^) and Tfh17 cell subset levels. At the same time, high-activity AS was linked to a negative correlation between total CM Tfh cells and serum level for IL-6 and IL-17F. It was revealed that amino acid homology between IL-17A and IL-17F comprises 50% but IL-17F is produced at an earlier stage of Th17 cell differentiation [81]. Moreover, it was shown that IL-17A stimulates ongoing immune response 10–30 times more readily than IL-17F [82]. Overall, it may be suggested that the correlation data account for the competitive relationships between the mechanisms underlying cellular and humoral immunity, being more pronouncedly evident in high-activity AS. Furthermore, only patients with high-activity AS were found to have decreased percentages of peripheral blood CM DP Tfh-cells.

While CM Th-cells subsequently differentiate into the EM subset, other features related to an altered Th-cell subset composition in AS patients were revealed. For instance, only low-activity AS was paralleled with a decreased count of total EM Th17 (CCR6^+^) cells, whereas high-activity AS was associated with elevated levels of Th2 cells. Furthermore, high-activity AS was associated with a positive relationship between peripheral blood Th2 cell level and serum IL-4 concentration. Such a relationship has been previously identified for this CM Th cell subset which accounts for humoral immunity and is involved in AS immunopathogenesis.

In investigating the EM Th17 cell subset composition in activity-driven AS patients, we detected similar changes. For instance, regardless of the disease activity, patients had elevated levels of classical and DN Th17 cells in peripheral blood. However, only high-activity AS patients showed a relationship between the Th17 cell subset composition and some serum cytokine levels, corroborating the role played by this cell subset in activating the cellular mechanisms underlying AS immunopathogenesis. In particular, a positive correlation between the level of classical Th17 cells and serum cytokine concentrations for IL-12(p40) and IFN-γ, as well as between total Th17 (CCR6^+^) cells and the DP Th17 subset, together with IFN-γ level, characterize the activation mechanisms for this Th-cell subset and its role in stimulating cell-mediated immunity in AS.

Finally, it is worth noting that the relationships observed for Th17 cells were observed solely in high-activity AS patients. Altogether, it was found that the AS disease course is characterized by an altered peripheral blood Th-cell phenotype in parallel with cell differentiation stage. Associations between certain serum cytokine or chemokine levels and percentage of any of the Th cell subsets examined in this study may suggest that they may be somehow linked, suggesting potential molecular or cellular targets for further interventions to alleviate AS, although this remains to be further investigated.

## 4. Materials and Methods

### 4.1. Patients

The study was conducted at the clinic of the Krasnoyarsk State Medical University from 2019 to 2021. Fifty-eight AS patients (14 females 44 males), aged 20–58 years old (Ме = 40.0 years [32.0; 47.0]), were enrolled. AS was verified based upon the New-York Criteria [83]. The inclusion criteria were disease onset over the age of 18 years and signed informed consent form. The exclusion criteria were as follows: the presence of other types of spondyloarthritis, osteoarthritis, acute and chronic exacerbated diseases; HIV infection; syphilis; immunodeficiency; hematological or oncological diseases; as well as other clinically significant conditions that may affect the results of the study. The inclusion criteria for healthy subjects was the absence of acute and chronic diseases. Disease activity was assessed using the BASDAI and ASDAS indices, adjusted to ESR and CRP levels according to the current nomenclature approved by the Assessment of SpondyloArthritis International Society and Outcome Measures in Rheumatology (ASAS/OMERACT) [84]. Based on disease-driven activity, all patients were stratified into two groups: absence or low activity AS (ASDAS-CRP < 2.1; n = 37) and high activity AS (ASDAS-CRP ≥ 2.1; n = 21). The clinical and laboratory patient characteristics with varying AS activity are shown in Table 9. Twenty-four AS patients received anti-TNFα (adalimumab—6 patients, infliximab—14, etanercept—3, golimumab—1) and 15 received anti-IL17 (secukinumab—8 patients, netakimab—7). All preparations were used at recommended doses and frequency of administration, with bDMARDs length comprising 1.5 [1.0; 4.5] years. Patients receiving standard therapy were treated with non-steroidal anti-inflammatory drugs (NSAID)—19 subjects (100%), glucocorticoids—13 subjects (68.4%), sulfasalazine—7 subjects (36.8%) and methotrexate—3 subjects (15.8%). Activity-driven AS patient stratification according to therapy intervention is shown in Table 10. The control group comprised 45 healthy volunteers (15 females and 30 males) aged 18–57 years old (Ме = 39.0 years [27.0; 47.0]). The study protocol was approved by the Ethics Committee at the Krasnoyarsk State Medical University (protocol 87/2018, dated 14/12/2018) and was conducted in accordance with the Declaration of Helsinki. Written consent was obtained from all participants.

**Limitations:** Due to the limited number of patients with high activity AS, the formation of the high activity group (ASDAS-CRP ≥ 2.1; n = 21) was problematic and led to significant differences in the numbers within the groups. However, the size of the groups represented the population of patients with AS in terms of age, gender and type of treatment from Krasnoyarsk region of Russian Federation.

### 4.2. Sample Collection

All experiments were performed within ≤6 h after blood collection. Fasting whole peripheral blood from every patient was collected in vacuum test tubes containing with K3-EDTA anti-coagulant. For cytokine measurement, cell-free plasma samples were obtained after whole blood centrifugation at 300 g for 7 min at +4 °C. Samples were then placed in new 1.5 mL tubes and centrifuged again at 300 g for 15 min at +4 °C to remove residual platelets and other blood cells. Finally, each plasma sample was aliquoted and stored at −80 °C until use.

### 4.3. Th Cell Flow Cytometry Immunophenotyping

**Figure 1:**
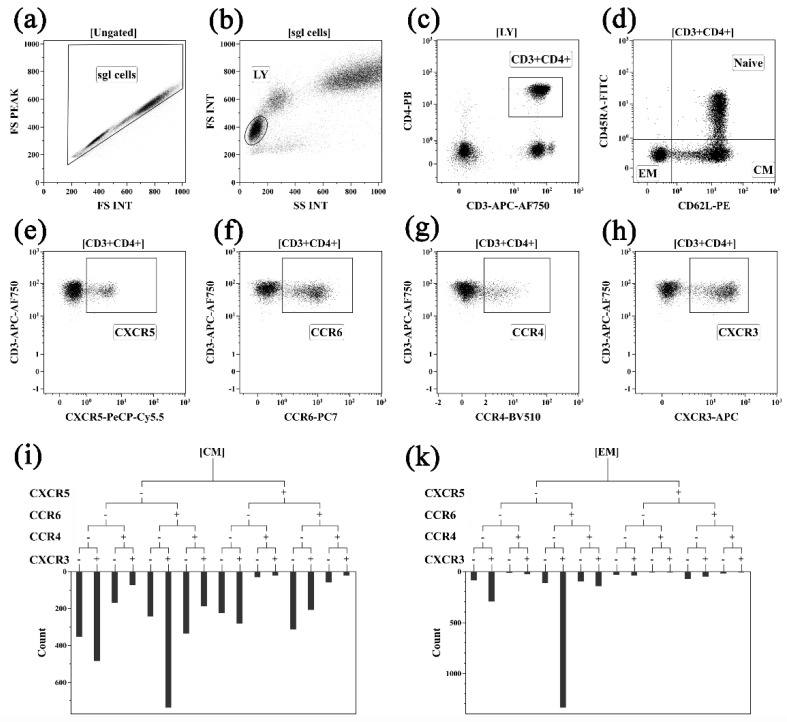
Flow cytometric gating strategy used to identify main Th cell subsets. (**a**) Singlet gating based on FS PEAK versus FS INT (the region is set to discriminate cell doublets); (**b**) Lymphocytes were gated on the side scatter/forward scatter plot with a “LY” gate; (**c**) Th cells were identified from the gate of lymphocytes as “CD3^+^CD4^+^” (**d**) and then separated into differentiated subsets using CD45RA and CD62L expression (‘naïve’ Th were CD45RA^+^CD62L^+^; central memory Th were CD45RA^–^CD62L^+^; effector memory Th were CD45RA^–^CD62L^–^ and “terminally differentiated CD45RA-positive” Th cells were CD45RA^+^CD62L^–^). Histograms (**e**–**h**)—expression of CXCR5, CCR6, CCR4 and CXCR3, respectively, by total Th cell population; regions “CXCR5”, “CXCR3”, “CCR6” and “CCR4” were used as branches for hierarchical tree histograms (**i**,**k**). Hierarchical tree histograms were gated on CM and EM Th—(**i**,**k**), respectively). The frequency histograms below the trees indicates the relative proportion of cells in each subset that expressed CXCR5, CXCR3, CCR6 and CCR4 within CM and EM Th.

An amount of 100 µL of whole blood was used for Th cell immunophenotyping by flow cytometry. Blood samples were stained with antibodies against CD3 (clone UCHT1) and CD4 (clone 13B8.2) to separate CD3^+^CD4^+^ T cells into distinct subsets using antibodies against CD45RA (clone 2H4LDH11LDB9 [2H4]) and CD62L (clone DREG56), based on a previously proposed gating strategy for major Th cell subsets [85]. Naïve Th cells were of phenotype CD45RA^+^CD62L^+^, whereas phenotypes CD45RA^–^CD62L^+^ and CD45RA^–^CD62L^–^ were identified as central memory (CM) and effector memory (ЕМ) T cells, and terminally-differentiated effector (TEMRA) CD45RA-positive Th cells were determined to be of the CD45RA^+^CD62L^–^ subset. CM and EM Th-cell subsets were further analyzed for chemokine receptor expression using additional marker-specific monoclonal antibodies: CCR4 (CD194, clone L291H4), CCR6 (CD196, clone G034E3), CXCR3 (CD183, clone G025H7) and CXCR5 (CD185, clone J252D4). Antibody staining was performed according to the manufacturer’s recommendations. A selection of optimal antibody combinations and relevant-antibody-conjugated fluorochrome combinations was performed according to a previously proposed protocol [86]. RBCs were lysed by adding ex tempore prepared 975 µL lysis buffer VersaLyse (Beckman Coulter, INpolis, IN, USA) to IOTest 3 Fixative Solution (Beckman Coulter, INpolis, IN, USA). After RBC lysis, the peripheral blood samples were washed once with excessive saline volume by centrifuging at 330 g for 7 min, followed by discarding sediment. Subsequently, cell pellets were resuspended in saline (pH 7.2–7.4) supplemented with 2% paraformaldehyde (Sigma-Aldrich, St. Louis, Missouri USA). Samples were analyzed using a Navios™ flow cytometer (Beckman Coulter, INpolis, IN USA) equipped with 405-, 488- and 638-nm diode laser modules. At least 50,000 lymphocyte events were analyzed in each sample. All flow cytometry data were assessed using Navios v.1.2 and Kaluza™ v.1.2 (Beckman Coulter, INpolis, IN, USA) software. The gating strategy and algorithms used for the Th cell subset analyses are shown on Figure 1; these were described previously in [85].

### 4.4. Multiplex Assay

Serum cytokine concentrations were measured with a MILLIPLEX^®^ MAP Human TH17 Magnetic Bead Panel (Merck, USA) in xMAP-based technology (Luminex, USA), according to the manufacturer’s recommendations. Data were collected and analyzed using a Luminex MAGPIX Instrument (Luminex, USA).

### 4.5. Statistical Analysis

Data samples were analyzed by calculating the median (Ме) and interquartile ranges (IQR), along with the 25th and 75th percentiles (С_25_–С_75_). Qualitative variables of clinical parameters were presented in absolute numbers and percentages (n (%)). A significance level for quantitative variables was assessed using a non-parametric Mann-Whitney U test. Qualitative variables were compared using the Fisher’s exact test. The strength of parameter relationships was assessed by calculating the Spearman rank R correlation coefficients. Statistical analyses were performed with the Statistica 8.0 software package (StatSoft Inc., 2007).

## 5. Conclusions

T helper (Th) cells are among the most numerous T lymphocyte fractions in cellular composition and account for various immunopathological conditions, including AS activity. It was found that patients with low-activity AS had reduced numbers of peripheral blood EM Th cells (CD45RA^–^CD62L^–^), whereas high disease activity was associated with decreased “naive” Th (CD45RA^+^CD62L^+^) cells and increased levels of CM Th cells (CD45RA^–^CD62L^+^). By analyzing Th cell surface chemokine receptor expression, we were able to determine the compositions of the CM and EM Th cell subsets. It was found that patients with high disease activity had decreased percentages of CM Th1 cells but increased level of CM Th2 lymphocytes. Only patients with high AS activity were found to have decreased numbers of CM DP Tfh cells, but decreased and increased levels of CM Tfh1 and Tfh2 cells were observed, regardless of the disease activity. Such changes in the CM Th cell subset composition primarily characterize the main AS immunopathogenetic mechanisms, which are initiated and enabled via the IL-23/IL-17 regulatory axis. Using multiplex analysis, we found increased serum levels of cytokines which stimulate the activity of cellular immunity (IL-12(p40), IL-17A, IL-17F, IFN-g and MSCF); notably, this did not appear to depend on disease activity. At the same time, in patients with serum high-AS activity, the concentrations of IL-5 were also increased, which accounts for the importance of the mechanisms underlying humoral immunity under the disease course. A correlation analysis between the CM Th cell subset composition and cytokine levels also confirmed the role of humoral immune mechanisms in high-activity AS. Because our examinations of AS patients were carried out during ongoing therapy, it may be assumed that traditional anti-inflammatory therapy and GIBT have a more pronounced effect on the mechanisms of cellular immunity, whereas the humoral immune response was less markedly inhibited. On the other hand, it can be assumed that the modality of AS course can be determined by the involvement of humoral immune reactions.

Subsequent differentiation of CM Th cells into EM Th cells was found to be associated with altered cell subset composition. Patients with high-activity AS had normalized Th1 cell counts but persistently increased level of Th2 lymphocytes, whereas only low disease activity was associated with persistent high levels of Th17 cells. Only in patients with high disease activity were changes in Th2 cell numbers related to altered IL-4 concentrations. In general, it can be concluded that Th cell subset differentiation in AS patients tends to change mainly at the CM Th cell stage; additionally, it is characterized by the IL-23/IL-17 regulatory axis but is associated with increased activity of the humoral immune mechanisms in cases of high-level disease. The presented features of Th cell differentiation characterize the features of immunopathogenesis depending on the activity of AS and should be taken into account when conducting immunotherapy.

## Figures and Tables

**Table 1:** Peripheral blood Th-cell subset composition in activity-driven AS patients (Ме (IQR)).

Parameters	Control	Low-Activity AS	High-Activity AS
Th cells, %	47.8 (44.9–52.7)	45.3 (40.1–49.4)	48.6 (40.2–53.0)
Th cells, cells/μL	775.7 (676.0–1053.2)	901.3 (735.3–1188.7)	1061.6 (726.4–1217.7)
‘Naïve’ Th, %	31.4 (25.7–40.1)	31.9 (23.6–43.5)	25.6 (18.7–33.3) р_1_ = 0.037
CM Th, %	41.5 (37.9–47.3)	45.4 (37.8–51.5)	54.0 (46.3–56.1) р_1_ < 0.001 р_2_ = 0.032
EM Th, %	22.9 (17.3–27.3)	18.5 (15.2–23.2) р_1_ = 0.048	18.8 (14.6–23.0)
TEMRA Th, %	0.59 (0.25–1.89)	0.86 (0.37–1.79)	0.78 (0.51–1.50)

Comments: Percentages of Th-cell subset composition are presented as proportions of the total Th cell population; р_1_—significant difference compared with the control group; р_2—_-//- patients with low-activity AS.

**Table 2:** Peripheral blood chemokine receptor-based CM Th-cell subset composition in activity-driven AS patients (Ме (IQR)).

Parameters	Control	Low-Activity AS	High-Activity AS
CM Th, cells/ μL	332.5 (266.8–468.8)	388.4 (331.5–496.0) р_1_ = 0.017	576.5 (337.3–687.5) р_1_ < 0.001
Th1 cells, %	12.96 (11.03–16.89)	10.89 (9.22–13.78)	11.36 (6.61–13.29) р_1_ = 0.035
Th2 cells, %	10.67 (9.29–12.98)	13.83 (9.83–15.83)	14.92 (10.99–18.08) р_1_ = 0.014
Th17 Th, %	35.78 (32.94–43.69)	40.98 (35.85–47.36) р_1_ = 0.007	40.41 (36.42–43.87) р_1_ = 0.048
follicular Th, %	18.89 (15.68–22.36)	18.89 (12.28–22.29)	17.65 (16.54–20.29)

Percentages of CM Th-cell subset composition are presented as proportions of the total CM Th cell population; р_1_—significant difference compared with the control group; р_2_— -//- patients with low-activity AS.

**Table 3:** Peripheral blood CM Th17-cell subset composition (%) in activity-driven AS patients (Ме (IQR)).

CM Th17 Cell Subsets	Control	Low-Activity AS	High-Activity AS
‘classical’ Th17	12.33 (10.35–15.69)	16.12 (13.39–17.58) р_1_ = 0.004	16.99 (14.62–18.55) р_1_ < 0.001
DN Th17	2.61 (1.72–3.16)	4.38 (3.46–5.37) р_1_ < 0.001	4.45 (3.56–6.07) р_1_ < 0.001
DP Th17	10.89 (8.58–12.65)	8.05 (7.20–10.31) р_1_ = 0.001	7.27 (5.70–10.39) р_1_ < 0.001
Th17.1	10.37 (8.16–12.74)	11.76 (8.63–15.54)	9.97 (6.52–13.13)

Percentages of CM Тh17-cell subset are presented as proportions of the total CM Th cell population; р_1_—significant difference compared with the control group.

**Table 4:** Peripheral blood CM Tfh-cell subset composition (%) in activity-driven AS patients (Ме (IQR)).

CM Tfh Cell Subsets	Control	Low-Activity AS	High-Activity AS
Tfh1	34.08 (29.87–36.40)	24.77 (21.82–28.69) р_1_ < 0.001	24.29 (21.71–30.06) р_1_ < 0.001
Tfh2	20.04 (18.20–24.45)	22.63 (19.53–28.17) р_1_ = 0.021	27.62 (20.92–33.03) р_1_ = 0.001
Tfh17	28.85 (26.71–35.16)	36.19 (30.68–40.48) р_1_ < 0.001	36.47 (31.56–39.46) р_1_ = 0.013
DP Tfh	13.72 (11.40–18.52)	14.09 (11.55–15.68)	11.34 (9.63–14.11) р_1_ = 0.015 р_2_ = 0.049

Percentages of CM Тfh-cell subset are presented as a proportion out of total CM Th cell population; р_1_—significant difference compared with the control group.

**Table 5:** Peripheral blood chemokine receptor-based EM Th-cell subset composition in activity-driven AS patients (Ме (С_25_–С_75_)).

Parameters	Control	Low-Activity AS	High-Activity AS
EM Th, cells/μL	178.0 (139.5–230.7)	159.7 (123.4–264.3)	205.1 (111.0–252.4)
Th1 cells, %	21.24 (16.11–29.20)	15.29 (13.12–26.35)	15.00 (11.98–25.13)
Th2 cells, %	1.35 (0.88–1.78)	1.64 (1.06–2.38)	2.02 (1.68–3.42) р_1_ < 0.001 р_2_ = 0.041
Th17 Th, %	52.32 (41.39–59.88)	58.84 (53.06–66.20) р_1_ = 0.040	58.48 (51.36–63.37)
follicular Th, %	8.10 (5.81–12.43)	7.68 (3.80–10.05)	9.21 (7.07–11.67)

Percentages of EM Tfh cell subsets are presented as proportions of the total EM Th cell population; р_1_—significant difference compared with the control group; р_2_—-//- patients with low-activity AS.

**Table 6:** Peripheral blood EM Th17 cell subset composition (%) in activity-driven AS patients (Ме (С_25_–С_75_)).

EM Th17 Cell Subset	Control	Low-Activity AS	High-Activity AS
‘classical’ Th17	13.01 (9.30–15.35)	15.63 (11.13–19.70) р_1_ = 0.035	19.16 (15.07–22.52) р_1_ < 0.001
DN Th17	2.33 (1.28–3.02)	3.52 (2.44–5.11) р_1_ = 0.001	3.97 (2.24–4.60) р_1_ = 0.009
DP Th17	11.44 (8.07–15.27)	11.31 (7.90–13.88)	10.48 (6.69–14.20)
Th17.1	24.02 (18.80–29.67)	25.00 (18.18–31.99)	22.11 (16.41–25.40)

Percentages of EM Тh17-cell subsets are presented as proportions of the total EM Th cell population; р_1_—significant difference compared with the control group.

**Table 7:** Peripheral blood EM Tfh-cell subset composition (%) in activity-driven AS (Ме (С_25_–С_75_)).

EM Tfh Cell Subsets	Control	Low-Activity AS	High-Activity AS
Tfh1	30.54 (23.90–34.47)	21.38 (17.11–27.08) р_1_ < 0.001	25.89 (18.91–30.03) р_1_ = 0.016
Tfh2	14.90 (10.34–18.45)	15.38 (11.96–20.60)	18.96 (13.64–26.33) р_1_ = 0.031
Tfh17	38.47 (32.33–43.07)	46.48 (40.91–48.40) р_1_ < 0.001	44.16 (35.08–46.68)
DP Tfh	16.20 (12.08–21.25)	16.14 (12.84–18.72)	13.12 (10.07–14.55) р_1_ = 0.019 р_2_ = 0.037

Percentages of EM Тfh-cell subsets are presented as proportions of the total EM Th cell population; р_1_—significant difference compared with the control group.

**Table 8:** Serum cytokine level (pg/mL) in activity-driven AS patients (Ме (С_25_–С_75_)).

Parameters	Control	Low-activity AS	High-activity AS
IL-1β	7.64 (5.01–17.93)	30.09 (17.97–59.97) р_1_ < 0.001	28.56 (13.51–39.94) р_1_ < 0.001
IL-2	0.44 (0.10–1.05)	1.05 (0.78–1.46) р_1_ = 0.016	0.98 (0.57–1.88)
IL-4	0.83 (0.24–1.92)	1.32 (0.72–2.65)	1.62 (0.87–3.80)
IL-5	3.80 (1.77–4.68)	4.24 (2.93–6.01)	6.38 (4.00–10.01) р_1_ = 0.017
IL-17A	2.22 (1.04–5.48)	6.17 (5.13–8.19) р_1_ = 0.003	5.48 (3.35–6.85) р_1_ = 0.048
IL-17E/IL-25	177 (99–308)	290 (207–450) р_1_ = 0.041	253 (216–344)
IL-17F	11.58 (11.58–18.54)	23.63 (17.53–45.82) р_1_ < 0.001	21.58 (15.53–46.90) р_1_ = 0.002
IFN-γ	10.64 (3.63–15.46)	16.75 (10.67–28.09) р_1_ = 0.026	18.73 (12.47–28.49) р_1_ = 0.021
TNF-α	19.12 (14.30–29.27)	29.27 (23.31–41.10)	22.41 (16.11–36.97)

р_1_—significant difference compared with control group.

**Table 9:** Patient clinical characteristics in activity-driven AS.

Parameters	Low-Activity AS	High-Activity AS	*р*
Age (years), Ме (С_25_–С_75_)	37 (30–45)	41 (39–48)	0.117
Gender, n (%) Females/Males	8 (21.6)/29 (78.4)	6 (28.6)/15 (71.4)	0.750
HLA-B27+, n (%)	29 (78.4)	19 (90.5)	0.301
Extra-skeletal signs, n (%)	13 (36.4)	7 (33.3)	1.000
BASDAI, Ме (IQR)	1.0 (1.0–2.0)	3.9 (2.6–4.9)	<0.001
ASDAS-CRP, Ме (IQR)	1.3 (1.1–1.6)	2.6 (2.1–3.6)	<0.001
BASFI, Ме (IQR)	1.0 (0.5–2.0)	3.9 (3.7–4.6)	<0.001
BASFI > 4, n (%)	0 (0)	10 (47.6)	<0.001
Leukocytes, 10^9^/L Ме (IQR)	6.94 (6.16–8.28)	8.08 (6.77–10.68)	0.094
Platelets, 10^9^/L Ме (IQR)	275.0 (23.7.0–318.0)	296.0 (248.0–332.8)	0.441
RBCs, 10^12^/L Ме (IQR)	4.88 (4.58–5.13)	4.76 (4.29–5.04)	0.454
Hemoglobin, g/L Ме (IQR)	14.60 (13.90–15.50)	13.70 (13.00–14.50)	0.052
CRP, mg/L Ме (IQR)	2.10 (0.79–3.08)	8.37 (3.71–21.82)	<0.001
ESR, mm/h Ме (IQR)	5.0 (4.1–6.9)	10.1 (5.2–21.9)	0.020

Quantitative data are presented by the median and interquartile ranges (median (IQR)). Qualitative variables are presented in absolute values and percentages (n (%)). Comparisons were made using the Mann-Whitney U test (for quantitative variables) and the Fisher’s exact test (for qualitative variables).

**Table 10:** Therapy-based patient distribution in activity-driven AS (n (%)).

Type of Therapy	Low-Activity AS	High-Activity AS	*р*
No bDMARDs	13 (35.2)	6 (28.6)	0.0502
Anti-IL17	9 (24.3)	6 (28.6)	0.4661
Anti-TNFα	15 (40.5)	9 (42.8)	0.1482

Qualitative variables are presented in absolute values and percentages (n (%)). Comparisons were made using the Fisher’s exact test. No bDMARDs—without biological disease-modifying anti-rheumatic drugs.

## Data Availability

The data generated and analyzed during this study are included in this published article. Additional information is available from the corresponding author on reasonable request.

## References

[1] Chen B., Li J., He C., Li D., Tong W., Zou Y., Xu W. (2017). Role of HLA-B27 in the pathogenesis of ankylosing spondylitis (Review). Mol. Med. Rep..

[2] Dahmani C.A., Benzaoui A., Amroun H., Mecabih F., Sediki F.Z., Zemani-Fodil F., Fodil M., Boughrara W., Mecheti B., Attal N. (2018). Association of the HLA-B27 antigen and the CTLA4 gene CT60/rs3087243 polymorphism with ankylosing spondylitis in Algerian population: A case-control study. Int. J. Immunogenet..

[3] Hu N., Liu D., Zhao N., Wang X., Bai Y., Sun H. (2021). Lack of association between TNF polymorphism and ankylosing spondylitis susceptibility in HLA-B27-positive population: A meta-analysis. Eur. Spine J..

[4] Bridgewood C., Sharif K., Sherlock J., Watad A., McGonagle D. (2020). Interleukin-23 pathway at the enthesis: The emerging story of enthesitis in spondyloarthropathy. Immunol. Rev..

[5] Chisălău B.A., Crînguș L.I., Vreju F.A., Pârvănescu C.D., Firulescu S.C., Dinescu Ș.C., Ciobanu D.A., Tica A.A., Sandu R.E., Siloși I. (2020). New insights into IL-17/IL-23 signaling in ankylosing spondylitis (Review). Exp. Ther. Med..

[6] Rezaiemanesh A., Abdolmaleki M., Abdolmohammadi K., Aghaei H., Pakdel F.D., Fatahi Y., Soleimanifar N., Zavvar M., Nicknam M.H. (2018). Immune cells involved in the pathogenesis of ankylosing spondylitis. Biomed. Pharmacother..

[7] Blair H.A. (2019). Secukinumab: A Review in Ankylosing Spondylitis. Drugs.

[8] Pedersen S.J., Maksymowych W.P. (2019). The Pathogenesis of Ankylosing Spondylitis: An Update. Curr. Rheumatol. Rep..

[9] Mauro D., Thomas R., Guggino G., Lories R., Brown M.A., Ciccia F. (2021). Ankylosing spondylitis: An autoimmune or autoinflammatory disease?. Nat. Rev. Rheumatol..

[10] Šućur A., Jajić Z., Ikić Matijašević M., Stipić Marković A., Flegar D., Lukač N., Kelava T., Kovačić N., Grčević D. (2020). Combined manual and automated immunophenotypisation identified disease-specific peripheral blood immune subpopulations in rheumatoid arthritis, ankylosing spondylitis and psoriatic arthritis. Clin. Exp. Rheumatol..

[11] Hayashi E., Chiba A., Tada K., Haga K., Kitagaichi M., Nakajima S., Kusaoi M., Sekiya F., Ogasawara M., Yamaji K. (2016). Involvement of Mucosal-associated Invariant T cells in Ankylosing Spondylitis. J. Rheumatol..

[12] Ciccia F., Guggino G., Rizzo A., Saieva L., Peralta S., Giardina A., Cannizzaro A., Sireci G., De Leo G., Alessandro R. (2015). Type 3 innate lymphoid cells producing IL-17 and IL-22 are expanded in the gut, in the peripheral blood, synovial fluid and bone marrow of patients with ankylosing spondylitis. Ann. Rheum. Dis..

[13] Mauro D., Macaluso F., Fasano S., Alessandro R., Ciccia F. (2019). ILC3 in Axial Spondyloarthritis: The Gut Angle. Curr. Rheumatol. Rep..

[14] Min H.K., Moon J., Lee S.Y., Lee A.R., Lee C.R., Lee J., Kwok S.K., Cho M.L., Park S.H. (2021). Expanded IL-22+ Group 3 Innate Lymphoid Cells and Role of Oxidized LDL-C in the Pathogenesis of Axial Spondyloarthritis with Dyslipidaemia. Immune Netw..

[15] Long S., Ma L., Wang D., Shang X. (2018). High frequency of circulating follicular helper T cells is correlated with B cell subtypes in patients with ankylosing spondylitis. Exp. Ther. Med..

[16] Wilbrink R., Spoorenberg A., Verstappen G.M.P.J., Kroese F.G.M. (2021). B Cell Involvement in the Pathogenesis of Ankylosing Spondylitis. Int. J. Mol. Sci..

[17] Subrahmanyam P.B., Maecker H.T. (2021). Mass Cytometry Analysis of T-Helper Cells. Methods Mol. Biol..

[18] Zhu J. (2018). T Helper Cell Differentiation, Heterogeneity, and Plasticity. Cold Spring Harb. Perspect. Biol..

[19] Li M., Zhou X., Zhou L., Yu Z., Fu L., Yang P. (2020). Meta-Analysis of Changes in the Number and Proportion of Regulatory T Cells in Patients with Ankylosing Spondylitis. Biomed. Res. Int..

[20] An H., Li X., Li F., Gao C., Li X., Luo J. (2019). The absolute counts of peripheral T lymphocyte subsets in patient with ankylosing spondylitis and the effect of low-dose interleukin-2. Medicine.

[21] Jiang Y., Yang M., Zhang Y., Huang Y., Wu J., Xie Y., Wei Q., Liao Z., Gu J. (2021). Dynamics of Adaptive Immune Cell and NK Cell Subsets in Patients With Ankylosing Spondylitis After IL-17A Inhibition by Secukinumab. Front. Pharmacol..

[22] Yang M., Lv Q., Wei Q., Jiang Y., Qi J., Xiao M., Fang L., Xie Y., Cao S., Lin Z. (2020). TNF-α inhibitor therapy can improve the immune imbalance of CD4+ T cells and negative regulatory cells but not CD8+ T cells in ankylosing spondylitis. Arthritis Res. Ther..

[23] Kwon O.C., Park J.H., Park M.C. (2021). Tumour necrosis factor inhibitor tapering in patients with ankylosing spondylitis at low disease activity: Factors associated with flare. Ther. Adv. Musculoskelet. Dis..

[24] Tsukazaki H., Kaito T. (2020). The Role of the IL-23/IL-17 Pathway in the Pathogenesis of Spondyloarthritis. Int. J. Mol. Sci..

[25] Deveci H., Turk A.C., Ozmen Z.C., Demir A.K., Say Coskun S.U. (2019). Biological and genetic evaluation of IL-23/IL-17 pathway in ankylosing spondylitis patients. Cent. Eur. J. Immunol..

[26] Liu D., Liu B., Lin C., Gu J. (2021). Imbalance of Peripheral Lymphocyte Subsets in Patients With Ankylosing Spondylitis: A Meta-Analysis. Front. Immunol..

[27] Mangare C., Tischer-Zimmermann S., Riese S.B., Dragon A.C., Prinz I., Blasczyk R., Maecker-Kolhoff B., Eiz-Vesper B. (2019). Robust Identification of Suitable T-Cell Subsets for Personalized CMV-Specific T-Cell Immunotherapy Using CD45RA and CD62L Microbeads. Int. J. Mol. Sci..

[28] Vanikova S., Koladiya A., Musil J. (2022). OMIP-080: 29-Color flow cytometry panel for comprehensive evaluation of NK and T cells reconstitution after hematopoietic stem cells transplantation. Cytometry A.

[29] Jameson S.C. (2021). The Naming of Memory T-Cell Subsets. Cold Spring. Harb. Perspect. Biol..

[30] Meraviglia S., Di Carlo P., Pampinella D., Guadagnino G., Presti E.L., Orlando V., Marchetti G., Dieli F., Sergi C. (2019). T-Cell Subsets (TCM, TEM, TEMRA) and Poly-Functional Immune Response in Patients with Human Immunodeficiency Virus (HIV) Infection and Different T-CD4 Cell Response. Ann. Clin. Lab. Sci..

[31] Al Barashdi M.A., Ali A., McMullin M.F., Mills K. (2021). Protein tyrosine phosphatase receptor type C (PTPRC or CD45). J. Clin. Pathol..

[32] Courtney A.H., Shvets A.A., Lu W., Griffante G., Mollenauer M., Horkova V., Lo W.L., Yu S., Stepanek O., Chakraborty A.K. (2019). CD45 functions as a signaling gatekeeper in T cells. Sci. Signal..

[33] Su Z., Huang D. (2021). Alternative Splicing of Pre-mRNA in the Control of Immune Activity. Genes.

[34] Sani M.M., Ashari N.S.M., Abdullah B., Wong K.K., Musa K.I., Mohamud R., Tan H.T. (2019). Reduced CD4+ terminally differentiated effector memory T cells in moderate-severe house dust mites sensitized allergic rhinitis patients. Asian Pac. J. Allergy Immunol..

[35] Golubovskaya V., Wu L. (2016). Different Subsets of T Cells, Memory, Effector Functions, and CAR-T Immunotherapy. Cancers.

[36] Johansen K.H., Golec D.P., Thomsen J.H., Schwartzberg P.L., Okkenhaug K. (2021). PI3K in T Cell Adhesion and Trafficking. Front. Immunol..

[37] Amarnani A.A., Poladian K.R., Marciano B.E., Daub J.R., Williams S.G., Livinski A.A., Hsu A.P., Palmer C.L., Kenney C.M., Avila D.N. (2020). A Panoply of Rheumatological Manifestations in Patients with GATA2 Deficiency. Sci. Rep..

[38] Dennehy K.M., Löll E., Dhillon C., Classen J.M., Warm T.D., Schuierer L., Hyhlik-Dürr A., Römmele C., Gosslau Y., Kling E. (2021). Comparison of the Development of SARS-Coronavirus-2-Specific Cellular Immunity, and Central Memory CD4+ T-Cell Responses Following Infection versus Vaccination. Vaccines.

[39] Matyushenko V., Isakova-Sivak I., Kudryavtsev I., Goshina A., Chistyakova A., Stepanova E., Prokopenko P., Sychev I., Rudenko L. (2021). Detection of IFNγ-Secreting CD4+ and CD8+ Memory T Cells in COVID-19 Convalescents after Stimulation of Peripheral Blood Mononuclear Cells with Live SARS-CoV-2. Viruses.

[40] Kasatskaya S.A., Ladell K., Egorov E.S., Miners K.L., Davydov A.N., Metsger M., Staroverov D.B., Matveyshina E.K., Shagina I.A., Mamedov I.Z. (2020). Functionally specialized human CD4+ T-cell subsets express physicochemically distinct TCRs. Elife.

[41] Scheu S., Ali S., Ruland C., Arolt V., Alferink J. (2017). The C-C Chemokines CCL17 and CCL22 and Their Receptor CCR4 in CNS Autoimmunity. Int. J. Mol. Sci..

[42] Yoshie O. (2021). CCR4 as a Therapeutic Target for Cancer Immunotherapy. Cancers.

[43] Lee A.Y., Körner H. (2020). CC chemokine receptor 6 (CCR6) in the pathogenesis of systemic lupus erythematosus. Immunol. Cell Biol..

[44] Meitei H.T., Jadhav N., Lal G. (2021). CCR6-CCL20 axis as a therapeutic target for autoimmune diseases. Autoimmun. Rev..

[45] Karin N. (2020). CXCR3 Ligands in Cancer and Autoimmunity, Chemoattraction of Effector T Cells, and Beyond. Front. Immunol..

[46] Levesque L.A., Roy S., Salazar N. (2021). CXCR3 Expression and Genome-Wide 3’ Splice Site Selection in the TCGA Breast Cancer Cohort. Life.

[47] Magnusen A.F., Rani R., McKay M.A., Hatton S.L., Nyamajenjere T.C., Magnusen D.N.A., Köhl J., Grabowski G.A., Pandey M.K. (2021). C-X-C Motif Chemokine Ligand 9 and Its CXCR3 Receptor Are the Salt and Pepper for T Cells Trafficking in a Mouse Model of Gaucher Disease. Int. J. Mol. Sci..

[48] Hsieh C.H., Jian C.Z., Lin L.I., Low G.S., Ou P.Y., Hsu C., Ou D.L. (2022). Potential Role of CXCL13/CXCR5 Signaling in Immune Checkpoint Inhibitor Treatment in Cancer. Cancers.

[49] Hussain M., Adah D., Tariq M., Lu Y., Zhang J., Liu J. (2019). CXCL13/CXCR5 signaling axis in cancer. Life Sci..

[50] Kazanietz M.G., Durando M., Cooke M. (2019). CXCL13 and Its Receptor CXCR5 in Cancer: Inflammation, Immune Response, and Beyond. Front. Endocrinol..

[51] Aldridge J., Ekwall A.H., Mark L., Bergström B., Andersson K., Gjertsson I., Lundell A.C., Rudin A. (2020). T helper cells in synovial fluid of patients with rheumatoid arthritis primarily have a Th1 and a CXCR3+Th2 phenotype. Arthritis Res. Ther..

[52] O’Neil T.R., Hu K., Truong N.R., Arshad S., Shacklett B.L., Cunningham A.L., Nasr N. (2021). The Role of Tissue Resident Memory CD4 T Cells in Herpes Simplex Viral and HIV Infection. Viruses.

[53] Pandya J.M., Lundell A.C., Hallström M., Andersson K., Nordström I., Rudin A. (2016). Circulating T helper and T regulatory subsets in untreated early rheumatoid arthritis and healthy control subjects. J. Leukoc. Biol..

[54] Wacleche V.S., Landay A., Routy J.P., Ancuta P. (2017). The Th17 Lineage: From Barrier Surfaces Homeostasis to Autoimmunity, Cancer, and HIV-1 Pathogenesis. Viruses.

[55] Kuwabara T., Ishikawa F., Kondo M., Kakiuchi T. (2017). The Role of IL-17 and Related Cytokines in Inflammatory Autoimmune Diseases. Mediators Inflamm..

[56] Schinocca C., Rizzo C., Fasano S., Grasso G., La Barbera L., Ciccia F., Guggino G. (2021). Role of the IL-23/IL-17 Pathway in Rheumatic Diseases: An Overview. Front. Immunol..

[57] Chatzileontiadou D.S.M., Sloane H., Nguyen A.T., Gras S., Grant E.J. (2020). The Many Faces of CD4+ T Cells: Immunological and Structural Characteristics. Int. J. Mol. Sci..

[58] Sehgal A., Irvine K.M., Hume D.A. (2021). Functions of macrophage colony-stimulating factor (CSF1) in development, homeostasis, and tissue repair. Semin Immunol..

[59] Sinha S.K., Miikeda A., Fouladian Z., Mehrabian M., Edillor C., Shih D., Zhou Z., Paul M.K., Charugundla S., Davis R.C. (2021). Local M-CSF (Macrophage Colony-Stimulating Factor) Expression Regulates Macrophage Proliferation and Apoptosis in Atherosclerosis. Arterioscler. Thromb. Vasc. Biol..

[60] Trus E., Basta S., Gee K. (2020). Who’s in charge here? Macrophage colony stimulating factor and granulocyte macrophage colony stimulating factor: Competing factors in macrophage polarization. Cytokine.

[61] Song J.E., Kim J.S., Shin J.H., Moon K.W., Park J.K., Park K.S., Lee E.Y. (2021). Role of Synovial Exosomes in Osteoclast Differentiation in Inflammatory Arthritis. Cells.

[62] Dankers W., den Braanker H., Paulissen S.M.J., van Hamburg J.P., Davelaar N., Colin E.M., Lubberts E. (2021). The heterogeneous human memory CCR6+ T helper-17 populations differ in T-bet and cytokine expression but all activate synovial fibroblasts in an IFNγ-independent manner. Arthritis Res. Ther..

[63] Den Braanker H., Razawy W., Wervers K., Mus A.C., Davelaar N., Kok M.R., Lubberts E. (2022). Characterizing memory T helper cells in patients with psoriasis, subclinical, or early psoriatic arthritis using a machine learning algorithm. Arthritis Res. Ther..

[64] Kotake S., Yago T., Kobashigawa T., Nanke Y. (2017). The Plasticity of Th17 Cells in the Pathogenesis of Rheumatoid Arthritis. J. Clin. Med..

[65] Leipe J., Pirronello F., Klose A., Schulze-Koops H., Skapenko A. (2020). Increased plasticity of non-classic Th1 cells toward the Th17 phenotype. Mod. Rheumatol..

[66] Van Hamburg J.P., Tas S.W. (2018). Molecular mechanisms underpinning T helper 17 cell heterogeneity and functions in rheumatoid arthritis. J. Autoimmun..

[67] Cerboni S., Gehrmann U., Preite S., Mitra S. (2021). Cytokine-regulated Th17 plasticity in human health and diseases. Immunology.

[68] Schinnerling K., Aguillón J.C., Catalán D., Soto L. (2017). The role of interleukin-6 signalling and its therapeutic blockage in skewing the T cell balance in rheumatoid arthritis. Clin. Exp. Immunol..

[69] Beurier P., Ricard L., Eshagh D., Malard F., Siblany L., Fain O., Mohty M., Gaugler B., Mekinian A. (2021). TFH cells in systemic sclerosis. J. Transl Med..

[70] Law H., Venturi V., Kelleher A., Munier C.M.L. (2020). Tfh Cells in Health and Immunity: Potential Targets for Systems Biology Approaches to Vaccination. Int. J. Mol. Sci..

[71] Kurata I., Matsumoto I., Sumida T. (2021). T follicular helper cell subsets: A potential key player in autoimmunity. Immunol. Med..

[72] Morita R., Schmitt N., Bentebibel S.E., Ranganathan R., Bourdery L., Zurawski G., Foucat E., Dullaers M., Oh S., Sabzghabaei N. (2011). Human blood CXCR5(+)CD4(+) T cells are counterparts of T follicular cells and contain specific subsets that differentially support antibody secretion. Immunity.

[73] Cui D., Tang Y., Jiang Q., Jiang D., Zhang Y., Lv Y., Xu D., Wu J., Xie J., Wen C. (2021). Follicular Helper T Cells in the Immunopathogenesis of SARS-CoV-2 Infection. Front. Immunol..

[74] Velu V., Mylvaganam G., Ibegbu C., Amara R.R. (2018). Tfh1 Cells in Germinal Centers During Chronic HIV/SIV Infection. Front. Immunol..

[75] Liu Y., Ji H., Zhao P., Yan H., Cai Y., Yu L., Hu X., Sun X., Jiang Y. (2019). Follicular helper T cell and memory B cell immunity in CHC patients. J. Mol. Med..

[76] Desbois A.C., Régnier P., Quiniou V., Lejoncour A., Maciejewski-Duval A., Comarmond C., Vallet H., Rosenzwag M., Darrasse-Jèze G., Derian N. (2021). Specific Follicular Helper T Cell Signature in Takayasu Arteritis. Arthritis Rheumatol..

[77] Weinstein J.S., Herman E.I., Lainez B., Licona-Limón P., Esplugues E., Flavell R., Craft J. (2016). TFH cells progressively differentiate to regulate the germinal center response. Nat. Immunol..

[78] Kudryavtsev I., Serebriakova M., Starshinova A., Zinchenko Y., Basantsova N., Malkova A., Soprun L., Churilov L.P., Toubi E., Yablonskiy P. (2020). Imbalance in B cell and T Follicular Helper Cell Subsets in Pulmonary Sarcoidosis. Sci. Rep..

[79] Cooper A.M., Khader S.A. (2007). IL-12p40: An inherently agonistic cytokine. Trends Immunol..

[80] Li Z., Huang Z., Bai L. (2021). Cell Interplay in Osteoarthritis. Front. Cell. Dev. Biol..

[81] Paiva I.A., Badolato-Corrêa J., Familiar-Macedo D., de-Oliveira-Pinto L.M. (2021). Th17 Cells in Viral Infections-Friend or Foe?. Cells.

[82] Zhao J., Lu Q., Liu Y., Shi Z., Hu L., Zeng Z., Tu Y., Xiao Z., Xu Q. (2021). Th17 Cells in Inflammatory Bowel Disease: Cytokines, Plasticity, and Therapies. J. Immunol. Res..

[83] Van der Linden S., Valkenburg H.A., Cats A. (1984). Evaluation of diagnostic criteria for ankylosing spondylitis. A proposal for modification of the New York criteria. Arthritis Rheum..

[84] Machado P.M., Landewé R., Heijde D.V., Assessment of SpondyloArthritis international Society (ASAS) (2018). Ankylosing Spondylitis Disease Activity Score (ASDAS): 2018 update of the nomenclature for disease activity states. Ann. Rheum. Dis..

[85] Golovkin A., Kalinina O., Bezrukikh V., Aquino A., Zaikova E., Karonova T., Melnik O., Vasilieva E., Kudryavtsev I. (2021). Imbalanced Immune Response of T-Cell and B-Cell Subsets in Patients with Moderate and Severe COVID-19. Viruses.

[86] Vaidyanathan P., Appleton E., Tran D., Vahid A., Church G., Densmore D. (2021). Algorithms for the selection of fluorescent reporters. Commun. Biol..

